# In Vitro and In Vivo Studies of IgG-derived Treg Epitopes (Tregitopes): A Promising New Tool for Tolerance Induction and Treatment of Autoimmunity

**DOI:** 10.1007/s10875-012-9762-4

**Published:** 2012-09-02

**Authors:** Leslie P. Cousens, Nader Najafian, Federico Mingozzi, Wassim Elyaman, Bruce Mazer, Leonard Moise, Timothy J. Messitt, Yan Su, Mohamed Sayegh, Katherine High, Samia J. Khoury, David W. Scott, Anne S. De Groot

**Affiliations:** 1 EpiVax, Inc., 146 Clifford Street, Providence, RI 02903 USA; 2 Brigham and Women’s Hospital, Boston, MA USA; 3 Children’s Hospital of Philadelphia, Philadelphia, PA USA; 4 McGill University Health Center-Montreal Children’s Hospital, Montreal, QC Canada; 5 Uniformed Services University of the Health Science, Bethesda, MD USA; 6 Institute for Immunology and Informatics, University of Rhode Island, Providence, RI USA

**Keywords:** Tregitopes, regulatory T cell epitopes, CD25^+^ FoxP3^+^ T cells, autoimmune disease, adeno-associated virus, hemophilia

## Abstract

Tregitopes are regulatory T cell epitopes derived from immunoglobulin G (IgG) that stimulate CD25^+^ FoxP3^+^ T cells to expand. In conjunction with these Tregs, Tregitopes can prevent, treat, and even cure autoimmune disease in mouse models, suppress allo-specific responses in murine transplant models, inhibit CD8^+^ T cell responses to recombinant adeno-associated virus (AAV) gene transfer vectors, and induce adaptive Tregs in DO11.10 mice. In this review of recent Tregitope studies, we summarize their effects in vitro and describe recent comparisons between intravenous IgG (IVIG) and Tregitopes in standard in vivo immune tolerance models. Further investigations of the mechanism of action of Tregitopes in the preclinical models described here will lead to clinical trials where Tregitopes may have the potential to alter the treatment of autoimmune disease, transplantation, and allergy, and to improve the efficiency of gene and protein replacement therapies.

## Introduction to Tregitopes

**Fig. 1:**
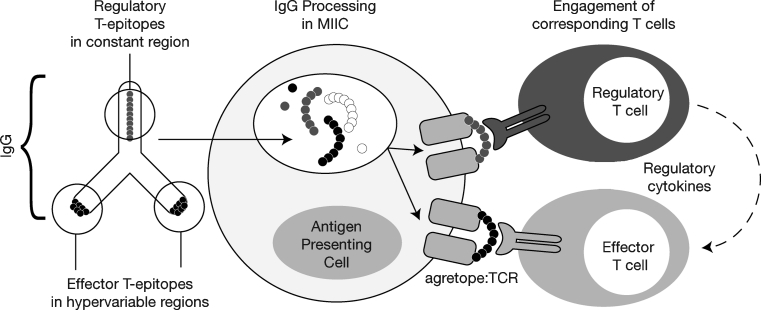
Tregitopes are highly conserved Treg epitopes found in IgG. They are postulated to reduce the immunogenicity of neo-epitopes in the hypervariable region of IgG CDR. De Groot AS, Moise L, McMurry JA, Wambre E, Van Overtvelt L, Moingeon P, et al. Activation of natural regulatory T cells by IgG Fc-derived peptide “Tregitopes.” Blood. 2008; 112(8):3303–11. American Society of Hematology, Copyright 2012. Reproduced with permission of American Society of Hematology (ASH)

In 2008 we discovered highly conserved, promiscuous T cell epitopes in immunoglobulin G (IgG) [1]. As these epitopes were highly conserved in the constant region of immunoglobulin G heavy chain (Fc) and immediately adjacent to the hypervariable domains of IgG (Fab)—in some cases the sequences were conserved in more than 90 % of human IgG sequences available for comparison in Genbank—we postulated that they might induce T cells that had a regulatory or immuno-suppressive function rather than an inflammatory function. Proof that natural regulatory T cell (nTreg) epitopes are present in immunoglobulin might explain why immunoglobulins, which undergo somatic hypermutation in the periphery, do not elicit the expected immune response against the new “foreign” hypervariable sequences. Studies summarized here provide evidence that the Tregitopes indeed provide inherent inhibitory signals. Moreover, Tregitope immunomodulatory signals that might counterbalance stimulatory signals from neo-epitopes expressed in immunoglobulin hypervariable regions (Fig. 1) might also be used to alter the course of autoimmune disease, allergy, and organ transplantation.

CD4^+^CD25^+^FoxP3^+^ nTregs are an important component of immune regulation in the peripheral circulation. Upon antigen-specific activation through their T cell receptor (TCR), nTregs suppress autoreactive T cell responses to unrelated antigens by both contact-dependent and -independent mechanisms [2]. Despite extensive studies, and with few exceptions [3, 4], the antigen specificity of nTregs is still unknown. The peptides we identified in IgG were tentatively named “IgG Tregitopes,” and we presented evidence that these peptides activate T cells that constitutively express FoxP3 and can be considered natural regulatory T cell epitopes. For the purpose of this discussion, we have defined Tregitopes as peptides that 1) bind to multiple major histocompatability (MHC) class II molecules, 2) induce nTregs to expand, 3) convert naïve and/or T effector cells to induced or adaptive Tregs (iTregs or aTregs), 4) suppress effector T cell responses to co-delivered antigen, and 5) up-regulate Treg-associated cytokines and chemokines.

In previously published work, we demonstrated that Tregitopes specifically activate CD4^+^CD25^+^FoxP3^+^ nTregs [1] and modify antigen-specific immune responses, as measured in tetramer-labeled cells in vitro. In further studies, we have demonstrated that co-incubation of antigens with Tregitopes leads to suppression of T and B effector responses, including suppression of antibody titers; reduced inflammatory cytokine secretion; and reduced proliferation, as measured by thymidine incorporation and CFSE dilution. Tregitope homologs that are nearly identical with human Tregitope sequences can be identified in mouse Fc and Fab; Tregitope homologs may also be present in murine albumin and other common murine proteins. The presence of murine T regulatory epitopes may explain earlier observations that Fc [5] and Fc-protein fusions stimulate tolerizing immune responses [6] in murine models.

**Fig. 2:**
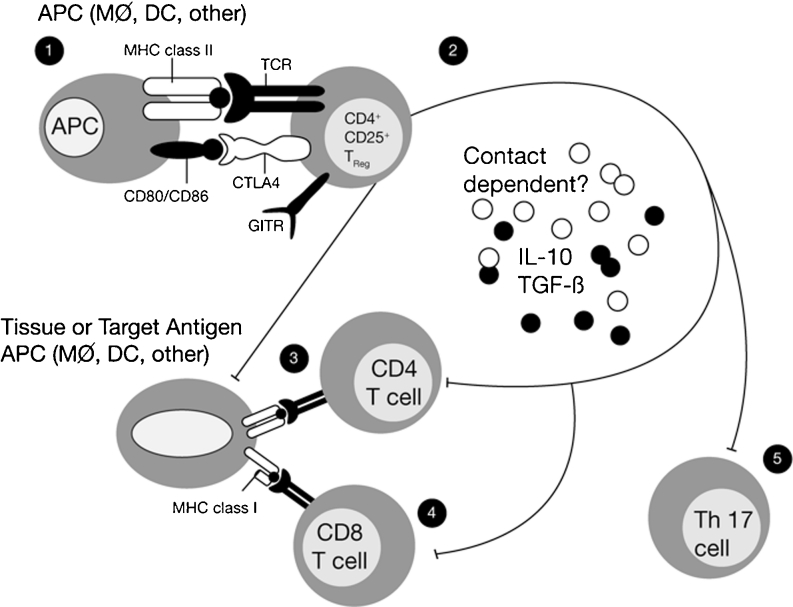
Proposed Tregitope mechanism of action. The following step-by-step sequence of events may lead to nTreg activation, antigen-presenting cell (APC) modulation, and either suppression of T effectors or induction of iTregs in the presence of Tregitopes: (Step **1**) Tregitopes are presented by activated APC to Tregs that have Tregitope-specific TCR. These nTregs then either indirectly through cytokine release (Step **2**), or directly suppress the activation of (Step **3**) antigen-presenting cells (macrophages, B cells, dendritic cells, or others), resulting in downregulation of the co-stimulatory signaling molecules CD80, CD86, and MHC class II. The cytokines released by the nTregs either directly affect (Steps **4** and **5**) the functioning of CD4 effector T cells, CD8 T cells, and Th17 T cells; or indirectly regulate (Step **3**) these cells through APC or direct cell-to-cell contact. Adapted from [2]

Induction of antigen-specific tolerance is the key to effective immunological therapy. In recent work, we have confirmed our initial observation that concurrent incubation or treatment with Tregitopes and antigen induces the conversion or expansion of iTregs [7]. However, once stimulated to respond, nTregs can modulate autoimmune responses not only by inhibiting the antigen-specific activity of autoreactive effectors, but also by changing the phenotype of the T effectors to that of an iTreg. We propose that the natural function of Tregitope-specfic T cells is to suppress anti-idiotypic responses (Fig. 1). Thus, activated Tregitope-specific nTregs recognize Tregitope peptides presented by antigen presenting cells (Fig. 2, Steps 1 and 2) and directly modulate antigen-presenting cells (Fig. 2, Step 3) and/or nearby effector T cells, leading to the conversion of these nearby CD4 and CD8 antigen-specific T effectors (Fig. 2, Step 4) to antigen-specific regulatory T cells (iTregs). This phenotypic change may be more or less transient and may require the preservation of a regulatory milieu and repeated treatment with Tregitopes and target antigen to allow iTregs to be maintained over longer periods of time.

## Relationship to IgG and IVIG

The role of antibodies in tolerance was described over a century ago [8], and the success of intravenous IgG (IVIG) therapy emphasizes the effectiveness of antibodies as immune regulators. The presence of Tregitopes in IgG may explain, in part, why IVIG therapy is efficacious in treating autoimmune diseases or in inducing tolerance in organ transplantation [9, 10]. The presence of natural T regulatory epitopes in IgG also may explain the effectiveness of IVIG as a therapy for factor VIII inhibitors (Bonn-Malmö protocol) [11, 12], for suppressing immunological rejection after transplantation (increased Treg suppression of graft-versus-host-disease [GVHD]) [13], and for allergic and autoimmune conditions [14, 15]. The identification and characterization of Tregitopes may be a novel and clinically important way to harness the utility of IVIG and apply it in a specific and quantitative manner.

IVIG’s effects have also been attributed to binding of the IgG Fc domain to Fc-gamma receptors, blockade of the FcRn receptor or CD95, and/or interaction of sialylated Fc with a dendritic cell lectin receptor SIGN-R1 [16, 17]. However, these models have never adequately accounted for the increase of Treg cells after IVIG treatment in psoriasis, skin transplants [18, 19], and experimental autoimmune encephalomyelitis (EAE) [20]. We propose that Tregitopes may be responsible for IVIG-induced tolerance; this hypothesis is corroborated by published reports that polyclonal immunoglobulin therapies induce expansion of Tregs and IL-10 secretion in vivo in animals and humans [21, 22].

The effect of IVIG on inflammation is achieved at amounts of IgG much higher than normally present in the blood. At these doses, multimeric complexes of IVIG may be formed, and the natural salvage pathway for IgG through FcRn (which normally protects IgG from degradation [23]) may be overwhelmed. Excess IgG and immune complexes are targeted for degradation in antigen-presenting cells such as macrophages and dendritic cells. Because we know that antigen associated with IgG immune complexes is processed and presented in the context of major histocompatibility complex (MHC) to T cells [24], we propose that degraded IgG in immune complexes may result in concurrent presentation of Treg epitopes that are contained within IgG.

In addition, the study of IVIG carried out by Anthony and Ravetch [25] indicates that IVIG contains some Ig molecules that have sialylated Fc, and that this fraction binds to SIGN-R1, a surface lectin receptor that targets sialylated proteins for degradation and antigen processing. Binding of the sialylated fraction of IVIG could be one means by which Tregitopes in IgG are internalized, processed, and presented in the context of MHC. However, sialylation of Fc does not appear to be an absolute requirement for tolerance induction by IVIG [26]. In fact, non-glycosylated peptide Tregitopes administered in saline have effects that recapitulate IVIG in vivo (see below). In addition, Tregitope effects are positively correlated with MHC binding, suggesting that the ultimate “receptor” for Tregitopes contained in IgG is MHC.

In addition to the Tregitopes identified in Fc, we have identified Tregitopes in the highly conserved framework regions of IgG Fab. A number of tolerogenic Fab-derived peptides have been described by other investigators [27–30]. Some of these peptides have been evaluated in clinical trials [30]. These peptides overlap with Fab-region Tregitopes, defined independently by our group, but are relatively MHC- and human leukocyte antigen (HLA)-restricted, based on EpiMatrix analysis. Independent confirmation of tolerogenic properties for these MHC-binding peptides by the Mozes laboratory provides evidence that Tregitopes are active in vitro and in vivo [30].

The five IgG-derived peptides described in Table I represent the best-characterized candidate Tregitopes that we have identified to date**.** These peptides were originally selected because of the presence of a high density of diverse MHC binding motifs (“promiscuity score”) and have been shown to bind promiscuously to human and murine MHC. Human and murine homologs of Tregitopes 167 and 289 have been studied extensively at EpiVax and by our collaborators; a high-level summary of recent results is provided in the following section. They are immunosuppressive in human ex vivo assays and, in their murine form, in BALB/c, C57BL/6, and NOD mice. Human Tregitopes 009 and 029 have been shown to suppress immune response in human ex vivo assays. The presence of certain Fab-region Tregitopes in licensed monoclonal antibodies is highly correlated with lower immunogenicity [31 and EpiVax unpublished data].

**Table I:** Five tregitopes found in IgG

Tregitope	Amino acids	Validation studies	# MHC motifs	Class II EpiMatrix promiscuity score (>10 = significant)	Location HC, LC
HTREG_IGGC-167	26	Published (De Groot et al. Blood 2008)	20	30.05	CH1
HTREG_IGGC-289	21	Published (De Groot et al. Blood 2008)	14	22.57	CH2
HTREG_IGGH-009	15	HLA-binding, Treg induction	7	14.07	VH
HTREG_IGGH-029	15	HLA-binding, Treg induction	7	14.09	VH
HTREG_IGGK-084	15	HLA-binding, Treg induction	7	16.38	VK

The five best-defined human IgG-derived Tregitopes listed above are 15 to 26 amino acids in length and have multiple HLA binding motifs and high EpiMatrix scores. These Tregitopes are located in the IgG constant domain (CH) and variable domains (VH, VK). Several mouse IgG homologs for these Tregitopes have been identified, although hTregitopes 167 and 289 bind to mouse MHC and, in a murine model of EAE, suppressed symptoms of EAE as well as IVIG [1, 31]

## Overview of Tregitope Research to Date

We have studied the effect of Tregitopes in several murine model systems. These include C57BL/6 and Balb/C mice (standard antigen immunization studies, using antigens such as ovalbumin (OVA); Scott, unpublished); NOD mice [7]; D011.10 TCR Tg mice (Su and Scott, unpublished); and bm12/ABM mice [32]. Tregitopes reduced the incidence of diabetes in NOD mice when administered prior to the development of diabetes; Tregitopes also suppressed the development of diabetes in a therapeutic model (treatment following two consecutive blood sugar levels exceeding 250 g/dL). In each of the models described above, Tregitope therapy effectively suppressed T cell responses in vivo. Suppression was measured in terms of cytokine expression, upregulation of nTreg and iTregs in vivo, and, in the case of bm12 into C57BL/6 mice, expansion of antigen-specific T cells.

Tregitopes have been administered with a variety of antigens. For example, the Najafian-Sayegh group has evaluated the mechanism of action in mixed lymphocyte reaction (MLR) studies in which the targets are alloantigens, as well as in vivo in models in which the target antigen is well defined (a single T cell epitope that is mismatched in bm12 to C57BL/6 skin transplant) and for which TCR transgenic mice are available (ABM) [32]. Mazer and Keegan have performed in vivo studies of Tregitope treatment in airway disease following induction of allergy in OVA-sensitized mice [33 and unpublished studies], Mingozzi and High have performed in vitro CD8^+^ T cell assays using both AAV and EBV antigens [34], and Elyaman and Khoury have administered Tregitopes with myelin oligodendrocyte glycoprotein (MOG) peptides in EAE [35].

Tregitope treatment effectively suppressed immune responses to the co-administered antigens in each of these systems, despite the varied nature of the antigens and the underlying bias of the immune response (Th1 in the case of MLR, Th2 in OVA, and Th17 in EAE). In each of these studies, concurrent treatment with a control peptide with similar MHC binding properties (tetanus toxin epitopes, influenza epitopes, and autologous MHC binding peptides such as GAD and insulin peptides) did not suppress immune response to the same degree as Tregitopes alone or Tregitopes administered with the target antigen. The effect of Tregitopes may be long-lasting in mice; we have observed effective immune suppression to alloantigens at 100 days in the transplant model [32] and reversal of diabetes for as long as 26 weeks in NOD mice [7].

Tregitopes’ mechanism of action has been the subject of extensive study. The current model is illustrated in Fig. 2, which also shows the immune system components that are believed to be involved in modulation of the immune response by Tregitopes. First, Tregitopes are recognized in the context of MHC by circulating Tregs (Fig. 2 [Step 1]). This leads to expansion and activation of the nTregs (Fig. 2 [Step 2]); IL-10 and TGF-beta are secreted and the phenotype of antigen-presenting cells is either directly or indirectly altered, leading to downregulation of co-stimulatory molecules (CD80, CD86, MHC class II) on the antigen-presenting cell surface and upregulation of tolerogenic factors such as ILT3 (Cousens et al., manuscript in preparation).

Suppression of co-stimulatory molecules leads to direct suppression of CD4 and CD8 T effector cells (Fig. 2 [Steps 3 and 4, Najafian and Mingozzi, unpublished studies]). Indirect effects may occur due to cytokines or direct contact between Tregs and T effectors (Fig. 2 [Steps 4 and 5]). Mingozzi and High have preliminary evidence that the nTregs directly contact CD8 T cells, modulating their responses in an antigen-specific manner (Mingozzi et al., manuscript in preparation; not shown in Fig. 2).

Due to the complex interplay of antigen-presenting cells, nTregs, T effector cells, and iTregs, the effects of Tregitope treatment in vitro and in vivo appear to be pleiotropic. This indicates that Treg responses to these peptides impact other cells central to the induction and maintenance of inflammation, e.g. antigen-presenting cells such as dendritic cells and, potentially, B cells. Thus, it is now somewhat less surprising to find that treatment with Tregitopes can suppress *humoral immune responses* to co-administered antigens, induce *adaptive T cells* (aTreg or iTreg), *suppress CD8* ^*+*^ *T cell* responses in an antigen-specific manner, suppress Th17 cells, and *suppress reactive airway responses* and IgE in an OVA model of allergy. Regardless of their site of action, Tregitopes are an attractive therapeutic intervention because they appear to modulate immune responses through natural networks, shifting the balance of immune responses to re-establish tolerance.

## Conclusions

We have studied Tregitopes and their activities in a range of model systems, proposed a mechanism of action of Tregitopes, and described their potential as modulators of tolerance in transplantation, gene therapy, and autoimmune disease. Information about Tregitopes present in monoclonals and Fc fusions has already had dramatic impact on the field of protein therapeutics. Indeed, a careful review of monoclonal antibody immunogenicity in clinical practice has already revealed a correlation between the presence of human Tregitopes and less immunogenic monoclonals [31], and the concept has been integrated into preclinical immunogenicity screening by a number of large biotech companies. Tregitopes also may be present in other proteins, as illustrated by studies carried out with albumin, a common excipient in protein therapeutics [36].

The use of Tregitopes is quite different from other emerging approaches for the induction of Tregs such as the anti-CD3 monoclonal antibodies teplizumab and otelixizumab. Anti-CD3 treatment has shown some efficacy in human studies, but the mechanism of Treg induction is elusive and the effect appears to be brief [37, 38]. Potential advantages to the Tregitope approach are induction of circulating Tregitope-specific nTregs, specificity of the tolerance to the co-administered antigen, and highly localized immunosuppressive effects. Induction of antigen-specific adaptive tolerance using Tregitopes that are co-delivered, linked, or fused to Tregitope peptides may be the key to treatment of autoimmune disease for which the target antigens have been identified; this would probably be effective for the treatment of allergies as well. Induction of tolerance using targeted Tregitope therapy may reduce the repeated and long-term treatments necessary to treat autoimmune diseases and allergies, if restoration of tolerance is achieved. Off-target effects generally associated with teplizumab and otelixizumab and other systemic immunosuppressive therapies (Cytoxan, prednisone) might be avoided if existing therapies for severe allergies and autoimmune diseases were replaced with or augmented by Tregitopes. However, maintaining tolerance induced by Tregitope therapy in humans may require “booster” treatments (Tregitopes and target antigen) and/or intermittent low-dose IL-2 to maintain iTreg populations [39].

Similarly, Tregitope therapy may also modulate pre-existing and de novo immune responses to adeno-associated virus (AAV) vectors, which are a major barrier to successful gene transfer. Integration of Tregitope into the AAV capsid has shown some promise in preliminary experiments conducted by Mingozzi et al., and induction of tolerance to AAV vectors could have wide-reaching effects for the gene transfer field. Finally, restoring natural tolerance to self or creating artificial tolerance to transplanted organs would alleviate the burden of repeated and long-term medical interventions required as part of the treatment for transplants.

As the discovery of Tregitopes may explain some effects of IVIG therapy, biomarkers of Treg activity may be important adjuncts to clinical trials of IVIG*.* Already, induction of IL-10, downregulation of CD154, and modification of surface expression of Notch and Jagged have been identified as potential markers of the Tregitope effect (Cousens, Elyaman, unpublished). Induction of nTregs specific for Tregitope may eventually be measurable in the clinical setting by staining CD4 T cells with Tregitope tetramers.

The duration and specificity of the Tregitope effect will be examined in ongoing safety studies. As the mechanism of action of Tregitopes closely parallels that of intravenous IgG (IVIG), a comparison with IVIG’s record of safety would appear to be appropriate. It is interesting to note that long term IVIG treatment has not been associated with increased cancer rates nor has it been associated with increased infections, thus we believe that Tregitopes will be relatively safe for long-term use. Dosing frequency is also under study; however, the effects of Tregitopes have been measured out to 26 weeks in the NOD model. If the antigen-specific effect of Tregitopes is due to the induction of relatively unstable antigen-specific Tregs, it is likely that Tregitope treatments will need to be repeated so as to maintain their effect. This may be an inconvenience, but it would also mean that Tregitope effects would be reversible. Thus we anticipate that Tregitopes will be safe and non-toxic and that their effects will be long-lasting but not permanent, unless a persistent form of co-expression with target antigen can be developed.

Future studies will elucidate key questions related to the duration of the Tregitope effect, clarify the number of nTregs that are Tregitope-specific, enable better understanding of the mechanism of Tregitope action, and uncover additional biomarkers of Tregitope activity. In addition, these studies will enable researchers to identify means of delivering Tregitopes directly to sites of inflammation, where it is presumed that they will induce nTregs to respond and suppress or convert allo- and/or auto-immune responses to adaptive tolerance.
